# Research on Chemically Deuterated Cellulose Macroperformance and Fast Identification

**DOI:** 10.3389/fpls.2021.709692

**Published:** 2021-10-01

**Authors:** Yan Song, Shaoyang Liu, Haoxi Ben, Yuanming Zhang, Guangting Han, Arthur J. Ragauskas, Wei Jiang

**Affiliations:** ^1^College of Textiles, Qingdao University, Qingdao, China; ^2^College of Textile and Clothing, Dezhou University, Dezhou, China; ^3^Department of Chemistry and Physics, Troy University, Troy, AL, United States; ^4^State Key Laboratory of Bio-Fibers and Eco-Textiles, Qingdao, China; ^5^Joint Institute of Biological Science, Biosciences Division, Oak Ridge National Laboratory, Oak Ridge, TN, United States; ^6^Department of Chemical and Biomolecular Engineering, University of Tennessee, Knoxville, Knoxville, TN, United States

**Keywords:** chemically deuterated cellulose, cotton, hydrogen-deuterium exchange, NIR, PLS

## Abstract

Chemically deuterated cellulose fiber was expected to provide novel applications due to its spectral, biological, and kinetic isotope effect. In this research, the performance of the chemically deuterated cotton fibers, including their mechanical property, enzymatic degradation performance, effect on bacterial treatment, and fast identification (near-infrared modeling) was investigated. The breaking tenacity of the deuterated cotton fibers was slightly lower, which might be attributed to the structural damage during the chemical deuteration. The glucose yield by enzymatic hydrolysis was less than that of the protonic cotton fibers, implying the deuterated fibers are less sensitive to enzymatic degradation. Furthermore, the deuterated fibers could promote the growth of bacteria such as *Escherichia. coli*, which was associated with the released low-level deuterium content. At last, the near-infrared technique combined with partial least squares regression successfully achieved a fast identification of the protiated and deuterated cotton fibers, which significantly promoted the potential application of deuterated cellulose as anticounterfeiting materials (e.g., special paper).

## Graphical Abstract

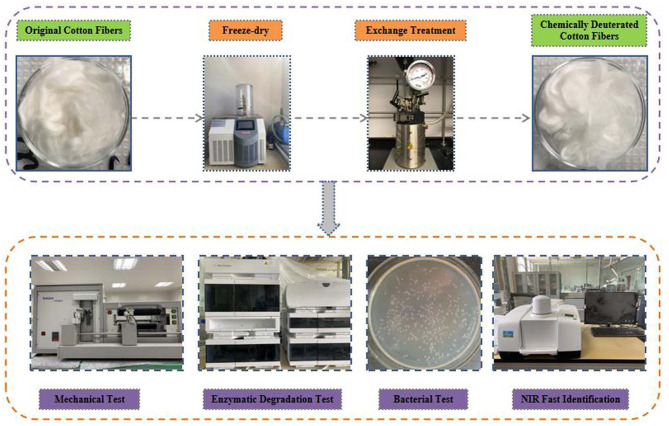

## Introduction

Cellulose is one of the most abundant and sustainable polysaccharides found on earth that has been extensively applied in fields such as biomaterials and biofuels (Ai et al., 2021). In addition, there are various modifications of cellulose materials, such as cellulose ester, cellulose ether, deuterated cellulose, etc., to expand the fields of cellulose application (Russell et al., 2015). As an important cellulosic modified product, deuterated cellulose, in which hydrogen atoms were replaced by deuterium atoms, could be produced through either biological or chemical means (Reishofer and Spirk, 2016). The biological mean involves the culture of plant or bacteria in a deuterated medium, which could both deuterate OH and CH with OD and CD, respectively. However, it usually takes a lot of time and effort, and also requires specific species (O'neill et al., 2015). Chemically deuterated fibers are produced by hydrogen–deuterium exchange treatment, which is rapid and easy to accomplish for a variety of cellulose materials with only OH replaced by OD (Tsuchikawa and Siesler, 2003). Therefore, the chemical mean is more frequently applied for cellulose deuterium incorporation.

Generally, chemically deuterated cellulose was applied in fundamental research like neutron studies because deuterium and hydrogen atoms interact with the neutrons very differently (Langan et al., 1999). The internal structure of cellulose-based materials (e.g., crystal structure and hydrogen bond parameter) that could not be detected by ordinary protiated samples was thus able to be uncovered. Meanwhile, deuterated cellulose materials are also expected to present spectral, biological, kinetic, and thermodynamic isotope effects (Reishofer and Spirk, 2016). These isotope effects alter the protiated cellulose performance and provide novel potential applications. Cotton fiber is one of the most important sources of cellulose (Haigler et al., 2012). Deuterated cotton fiber is not only easy to produce but also a good representative of various cellulose materials. A study on the performance of chemically deuterated cotton fibers is desired to expand the application field of cellulosic materials.

Near-infrared (NIR) spectroscopy provides fast quantitative and qualitative analyses with advantages of simple operation, low cost, zero-sample consumption, etc. (Wei et al., 2016). It has been successfully employed to identify protiated cellulose samples from different sources, such as different fibers, fabrics, and woods (Wei et al., 2016; Zhou et al., 2018). As previously reported by our group, deuterated cellulose fibers had conspicuous spectral isotope effects in the infrared (IR) and NIR regions (Song et al., 2021). If a simple NIR method could be established to distinguish chemically deuterated and protonic cellulose fibers, the deuterated fibers could be employed as an anticounterfeiting material. NIR analysis is often combined with different pattern-recognition methods, such as soft independent modeling of class analogy (SMICA), principal component regression (PCR), and partial least squares regression (PLS). SMICA is a supervised discriminant analysis method, and PCR/PLS are both data description and dimension reduction method (Li et al., 2020). All the methods had been successfully used to classify or identify plant fibrous materials (Wei et al., 2016).

In this research, cotton fibers were chemically deuterated at different deuteration levels and their performance, including mechanical property, biological property, and enzymatic hydrolysis property, was characterized. Then, NIR coupled with SMICA, PCR, or PLS was investigated to build a fast and nondestructive method to detect deuterated fibers.

## Materials and Methods

### Materials

Cotton fibers were obtained from Dezhou Hengfeng Co., Ltd. Chemical reagents were purchased from Sigma Aldrich and used as received. The D_2_O applied was 99.99% in purity.

### Production of Chemically Deuterated Cellulose Fiber

The cotton fiber samples were first immersed in D_2_O and frozen-dried. Then, they were chemically deuterated with D_2_O (1:30, w/v) in a 4560 series Parr reactor (Parr Instrument Company, USA). The cotton fibers and D_2_O were heated to pre-determined temperature (from 25 to 205°C with a step of 10°C) and reacted for 8 h. Alkaline catalyst, K_2_CO_3_ or NaOH, was added to adjust the deuteration rate. After the hydrogen–deuterium exchange treatment, the deuterated fibers were filtered and washed with D_2_O until the effluent was pH neutral. Next, the deuterated fibers were dried at 60°C in a vacuum oven for 24 h. The dried deuterated fibers were stored at constant temperature and humidity (25 ± 2°C, 65 ± 5% RH, H_2_O moisture) for at least 2 weeks before analysis.

### Deuteration Rate Analysis by Fourier-Transform Infrared Spectroscopy (FTIR)

Fourier-transform infrared spectroscopy spectra of the chemically deuterated fibers were recorded with a PerkinElmer Spectrum 100 FTIR-ATR spectrometer (Waltham, MA, USA). The spectra in the wavelength range of 800–4,000 cm^−1^ were collected with a resolution of 2 cm^−1^ and a scan number of 64. The deuteration level of the fibers was calculated by the peak intensity ratio of OH/(OH+OD) after baseline correction, as previously reported (Song et al., 2020).

### Breaking Tenacity of the Fibers

Breaking tenacity was chosen as a representative of the mechanical properties of the fibers in this study. An FAVIMAT AIROBOT automatic single-fiber-strength tester was employed to measure the breaking tenacity of the cotton fibers, according to Chinese national standard GB/T5886-1986 “Experimental method of ramie single fiber breaking strength.” The original cotton fibers, cotton fibers treated by D_2_O (or H_2_O), and 10% (by weight) K_2_CO_3_ at 25°C (FTIR deuteration rate: 15 ± 3%), 65°C (FTIR deuteration rate: 25 ± 3%) and 105°C (FTIR deuteration rate: 35 ± 3%) were analyzed. For each sample, 50 single fibers were tested and the average value was recorded.

### Enzymatic Hydrolysis of the Fibers

The cotton fibers that were chemically deuterated by D_2_O (or H_2_O) with 10% K_2_CO_3_ at 65, 105, 145°C (FTIR deuteration rate: 45% ± 3%), and 185°C (FTIR deuteration rate: 55 ± 3%) were chosen as representative samples to investigate the enzymatic hydrolysis performance of the deuterated fibers.

The cotton fiber samples were first cut into small pieces to pass a 100-mesh sieve. Then, they were filtered with a 120-mesh sieve to obtain fine cotton fiber particles. Next, 0.1 g of fiber sample was suspended in 40 ml of citric acid salt buffer (0.1 mol/L, pH 4.8) and 1% (v/v) antibiotic and antifungal tertiary antibody solution. Novozymes Cellic CTec 2 enzyme with a filter paper activity of 0.147 filter paper unit (FPU)/mg was used in this study. The enzyme was a mixed enzyme that could hydrolyze the 1,4-β-d-glucosidic bonds of cellulose and other β-d-glucans. The amount of Cellic CTec 2 enzyme added was 20 FPU/g dextran cellulase (Wang et al., 2004, 2006). The above mixture was continuously oscillated at 50°C for 21 days to hydrolyze the cotton fibers. Then, the reaction was quenched with a boiling water bath for 10 min. The sample was centrifuged at 12,000 rpm for 20 min. The supernatant was separated and frozen at −20°C until glucose analysis.

The glucose content in the supernatant was quantified with an Agilent 1,260 Infinity II HPLC equipped with a Agilent PolarGel-M column (25 cm × 2.0 mm × 5 μm) and an electrochemical detector. The supernatant was filtered with a 0.2-μm syringe filter before injection. The injection volume was 0.5 mL, the mobile phase was tetrahydrofuran, the column temperature was 50°C, and the flow rate was 0.40 mL/min. Glucose standard solutions with concentrations of 0.20, 1.0, 4.0, and 12 g/L were used for calibration.

### Antibacterial Effect of the Deuterated Fibers

The cotton fibers treated with D_2_O (or H_2_O) and 10% K_2_CO_3_ at 65, 105, 145, and 185°C were chosen as representative samples to study the antibacterial effect of the deuterated fibers. The Chinese National Standard GB/T 20944.3-2007 “Textiles: Evaluation of antibacterial properties. Part 3: Oscillation method” was employed. A Gram-negative strain of *E. coli* (ATCC 11229) was used in this work. The bacteria were cultivated for 4 h to evaluate the effects.

In addition, the concentration of deuterium in the bacterial culture medium was determined by ^2^H-NMR, with DMSO-d6 as the external standard. The NMR analyses were performed on a Bruker Avance-III HD 400 MHz spectrometer operating at a frequency of 400.17 MHz. The ^2^H-NMR experiments were performed at a 9.45-μs (90°) ^2^H pulse, 5-s recycle delay, and 256 scans. The deuterium concentration in the culture medium was calculated with the ratio of the deuteron peak areas and moles of deuteron from DMSO-d6 and HOD (partially deuterated water), respectively.

### NIR Fast Identification of the Deuterated Fibers

Near-infrared identification models were studied to quickly distinguish the chemically deuterated cellulose fibers from common protonic fibers. There were 110 deuterated fibers and 40 controlled protonic fiber samples. Their deuteration rates were evenly distributed in 1–10, 10–20, 20–30, 30–40, 40–50, and 50–60% regions, respectively. The deuterated fibers were further classified as low (1–20%) and high (20–60%) deuteration level fibers, respectively. NIR spectra of the fibers were collected using a PerkinElmer Frontier spectrometer with a wavenumber range of 10,000–4,000 cm^−1^, resolution of 4 cm^−1^, and a scan number of 32. The control, low, and high deuterate rate samples were modeled and verified with SMICA and PRC/PLS methods, respectively. Concretely, 50 deuterated samples (20%~60%) and 30 protonic samples were used for modeling to identify high deuteration rate fibers. Another 20 high deuteration rate samples and 10 protonic samples were employed for verifying. When involving the low deuteration rate samples, 80 deuterated samples (1–60%) and 30 protonic samples were used for modeling. Then, additional 30 deuterated samples and 10 protonic samples were applied for verifying. The NIR identification models were established step by step for optimal identification results.

## Results and Discussion

### Deuteration of the Fibers

As previously reported by our group, after chemical deuteration treatment, the FTIR spectra presented the OD characteristic peak (2,500 cm^−1^). The FTIR spectra after baseline correction were applied to calculate the deuteration rate [OD/(OD+OH)]. It was also reported that deuteration rate could be controlled by adjusting the treatment temperature, time, catalyst, etc. (Song et al., 2020). Certain examples are presented in Table 1 to show the deuteration rates of the fibers under different treatment conditions (Song et al., 2021).

**Table 1:** Deuteration rate of the deuterated fibers (X°C-fibers treated at X°C).

**Samples**	**25°C**	**65°C**	**105°C**	**145°C**	**185°C**
Deuteration rate (no catalyst)/%	9.78 ± 1.98	15.31 ± 2.09	22.61 ± 3.01	25.36 ± 3.17	30.84 ± 3.66
Deuteration rate (with catalyst)/%	18.24 ± 2.35	25.49 ± 3.15	35.35 ± 3.52	45.98 ± 3.89	55.99 ± 3.24

### Breaking Tenacity of the Chemically Deuterated Cellulose Fibers

**Figure 1:**
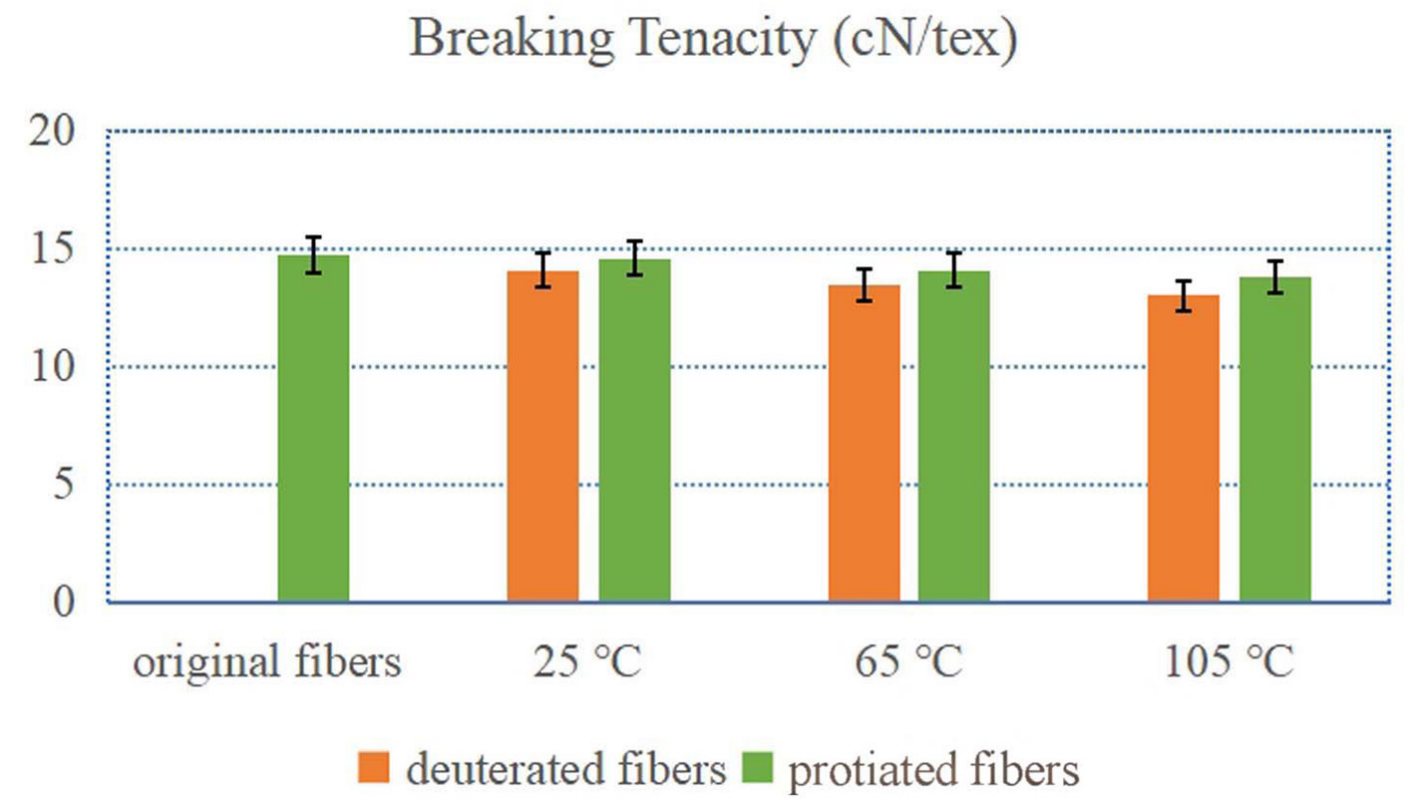
Breaking tenacity of deuterated cotton fibers and protonic cotton fibers (X°C-fibers treated at X°C).

Figure 1 exhibited the breaking tenacities of the deuterated and control protonic fibers before and after the exchange treatments. When treated at 25°C, the breaking tenacities of both the deuterated and control fibers decreased slightly (from 14.74 cN/tex to 14.08 cN/tex and 14.58 cN/tex, respectively, FTIR deuteration rate of 15% ± 3%). With the increase of treating temperature, decreases in the breaking tenacity of the fibers became larger. The treatments with higher temperatures could lead to more severe damages to the microstructure of the cotton fibers which could lower their breaking tenacities. It can also be noted that the breaking tenacities of the deuterated fibers were smaller than those of the corresponding control fibers. Although the exchange of deuterium enhanced the hydrogen bond, D_2_O could more seriously damage the microstructure of the cotton fibers than H_2_O during the treatment (Bolvig et al., 2000). In general, the effect of the hydrogen–deuterium exchange treatment on the breaking tenacity was limited. The breaking tenacity of the fiber only reduced less than 10% when treated at 105°C with D_2_O (FTIR deuteration rate of 35 ± 3%).

### Isotope Effect on Enzymatic Hydrolysis of the Deuterated Fibers

**Figure 2:**
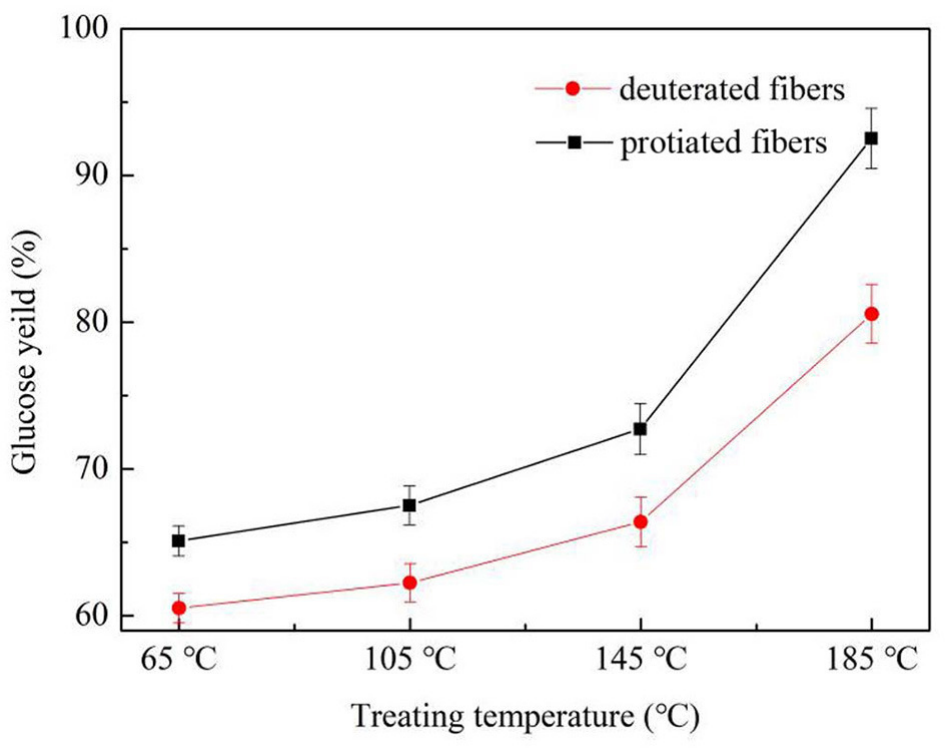
Enzymatic hydrolysis glucose yield of deuterated cotton fibers and protonic cotton fibers (X°C-fibers treated at X°C).

The isotope effect of the enzymatic hydrolysis performance on the fibers is illustrated in Figure 2. After the 21-day enzymatic hydrolysis, the glucose yield of the fibers increased with the increase of treating temperature. The decrease in stability after the higher-temperature treatments was due to the heat damage of the fibers, which had led to more enzyme contact areas. Furthermore, the deuterated fibers were hydrolyzed less than the protonic ones under all treatment temperatures. This phenomenon is known as the kinetic isotope effect (KIE). The deuterium-incorporated cotton fibers possessed higher binding energy and shorter bond length of deuterium bonds compared with hydrogen bonds, leading to slower reaction rates (Bhagia et al., 2018). Also, with the increase of treating temperature, the deuteration rate of the deuterated fibers rose, resulting in more deuterium bonds. So, a bigger glucose yield gap between the deuterated and protonic fibers was observed. In summary, the deuterated fibers were more stable to enzymatic degradation than the protiated ones.

### Antibacterial Effect of the Deuterated Fibers

**Figure 3:**
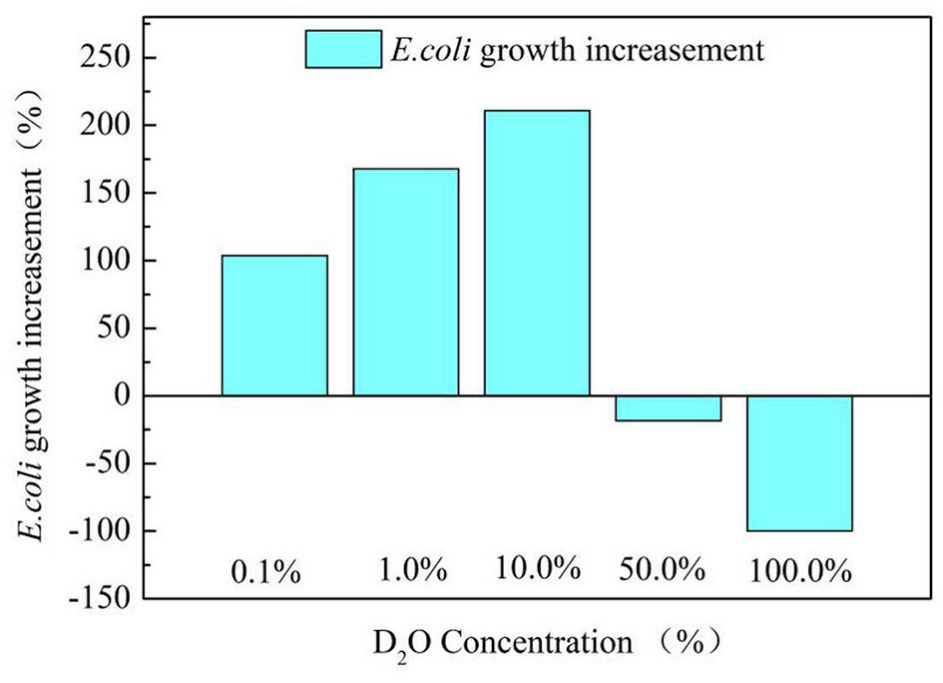
*Escherichia coli* growth situation at different D_2_O concentration culture.

Figure 3 exhibits the *E.coli* growth situation at different D_2_O concentrations in the culture medium. Deuterium concentration could affect the growth of *E.coli* differently. Very high deuterium concentration (such as 50% or 100%) had an adverse impact on the *E.coli* growth because the bacteria could not adapt to the high deuterium environment quickly. However, low deuterium concentration in the medium (such as 1% or 0.1%) promoted the growth of *E.coli*, which is consistent with previous reports (Anthony, 2013; Xueshu et al., 2014).

Table 2 shows the effect of the deuterated fibers on the growth of *E.coli*. The bacteria grew quicker in the culture medium with deuterated fibers by the oscillation method. The bacteria cultured with the deuterated fibers increased from 12.6 to 58.8% compared with those cultured with the original protonic cotton fiber. The higher deuteration rate of the fiber, the larger increase was observed.

**Table 2:** Bacterial increase when cultured with the deuterated fibers (X°C-fibers treated at X°C-with catalyst).

**Samples**	**65°C**	**105°C**	**145°C**	**185°C**
Bacterial increase /%	12.6	31.7	40.2	58.8
Deuterium content in culture medium /%0	0.16	0.17	0.19	0.23

The bacterial increase was related to the deuterium content in the culture medium. As shown in Table 2, the deuterium contents in the medium with deuterated fibers ranged from 0.16 to 0.23%0 after a 4-h culture, which were higher than that in the medium with ordinary protonic fiber (0.15%0). The additional deuterium was released dynamically by the deuterated fibers. As mentioned above, the low-level deuterium enrichment promoted bacterial growth. This behavior could be explained by both hormesis as well as the isotopic resonance hypothesis (Anthony, 2013; Xueshu et al., 2014). However, it begs for additional verification by more precise measurements. The deuterated fibers may be used to promote cultures of certain beneficial bacteria.

### The NIR Fast Identification of the Deuterated Fibers

The fast identification of deuterated cellulosic fiber using SMICA, PCR, and PLS methods was investigated in this research. SMICA models exhibit their identification result by recognition rate and rejection rate, which refer to the number of samples in and out of the area of the models. Prior to modeling, the original NIR spectra were baseline corrected and pretreated using the Savitzky–Golay first derivative (SG 1st-Der) to minimize the influence of baseline drift, reduce noise, and enhance the spectral characteristic features. The identification models were optimized step by step by adjusting model parameters, pretreatments, and spectral ranges.

As shown in Table 3, the best SMICA model distinguished the high deuteration rate (20–60%) and protonic fibers very well. All the recognition and rejection rates for both the training and verification sets were higher than 97%. However, when the low deuteration rate (1%~20%) fibers were added, the performance of the SMICA model dropped significantly, especially for the verification set. The recognition and rejection rates were as low as 72 and 90%, respectively. Thus, the SMICA model could only be used to distinguish the protonic and high deuteration rate fibers.

**Table 3:** Classification result of soft independent modeling of class analogy (SMICA) models [A: high deuteration rate (20–60%) fibers and protonic fibers; B: high and low deuteration rate (1–60%) fibers and protonic fibers].

	**Sample**	**Protonic cotton fibers (Calibration set)**	**Deuterated cotton fibers (Calibration set)**	**Verification set**
A	Recognition rate (%)	100 (30/30)	100 (50/50)	97
	Rejection rate (%)	100 (50/50)	97 (29/30)	100
B	Recognition rate (%)	100 (30/30)	100 (80/80)	72
	Rejection rate (%)	97 (78/80)	83 (25/30)	90

Both PCR and PLS models first assign different samples with different values (1, 2, 3, … *n*). Then, the models are evaluated by whether the predicted value is inside the ± 0.5 error range of the assigned values.

**Figure 4:**
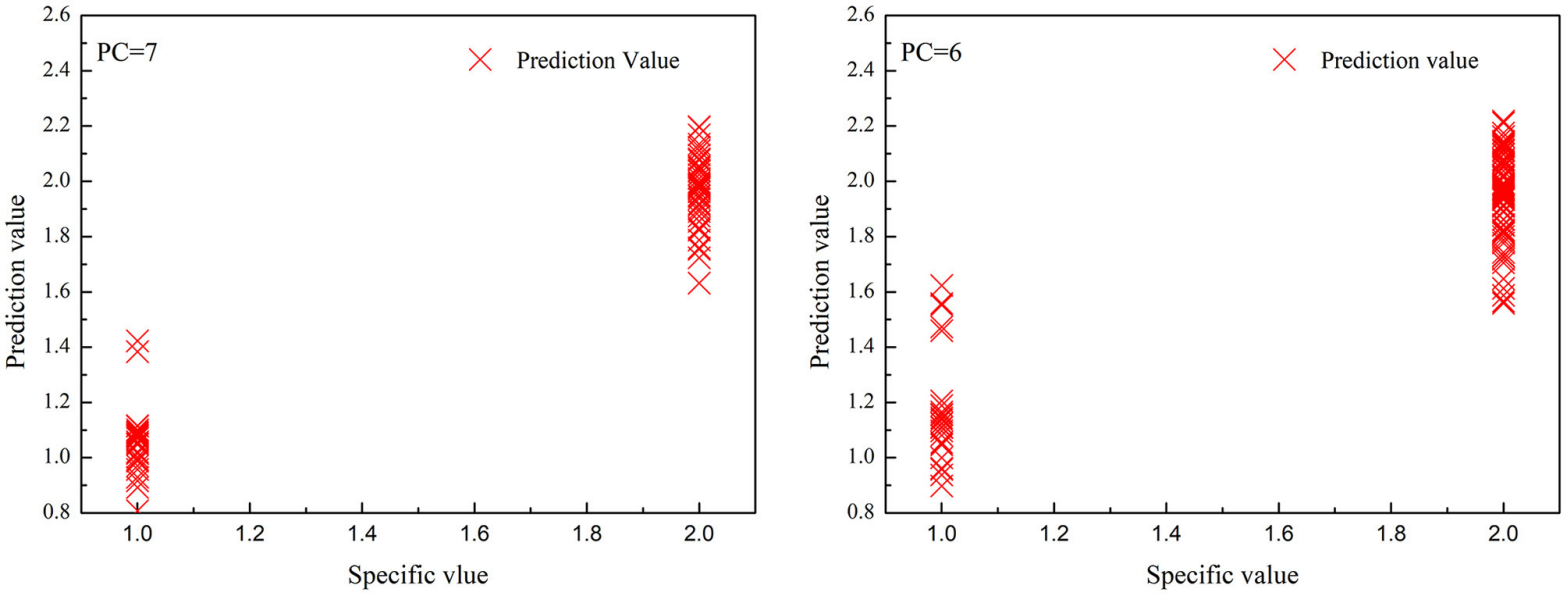
The correlations between predicted value and specified value for principal component regression (PCR) models in the prediction set. (left): high deuteration rate (20–60%) fibers and protonic fibers; (right): high and low deuteration rate (1–60%) fibers and protonic fibers.

**Figure 5:**
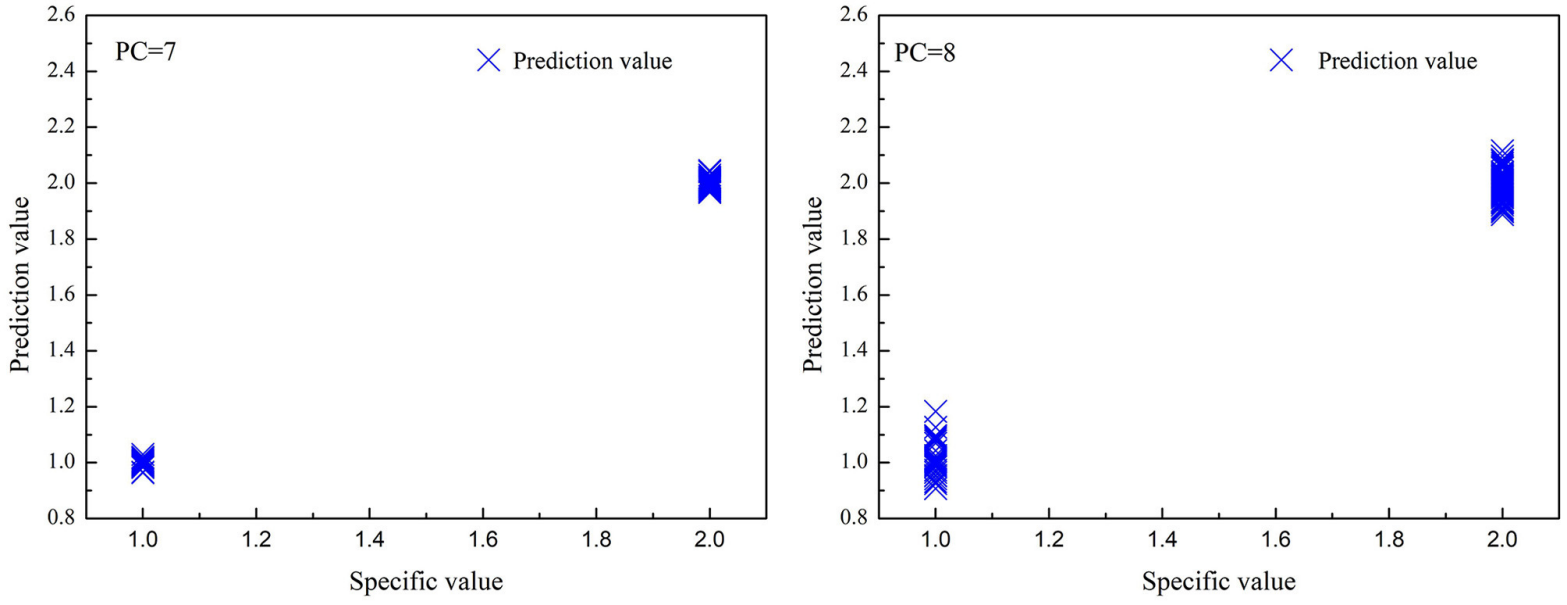
The correlations between predicted value and specified value for partial least squares regression (PLS) models in the prediction set. (left): high deuteration rate (20–60%) fibers and protonic fibers; (right): high and low deuteration rate (1–60%) fibers and protonic fibers.

Tables 4, 5 exhibit the PCR and PLS model identification results, respectively. Figures 4, 5 depict the correlations between the predicted and specific values for the PCR and PLS models in the prediction set, respectively. As shown in Table 4, the best PCR models also distinguished the high deuteration rate and protonic fibers nicely. The recognition and rejection rates were as high as 100 and 90%, respectively. However, the recognition rate lowered to 70%, and the predicted values scattered much wider when both the high and low deuteration rate fibers were included in the model, as can be seen from Figure 4. They suggest that the PCR models could not effectively identify the chemically deuterated fibers when the deuteration rate was lower than 20%.

**Table 4:** Classification result of principal component regression (PCR) models [cross-validation, A: high deuteration rate (20–60%) fibers and protonic fibers; B: high and low deuteration rate (1–60%) fibers and protonic fibers].

**Sample**	**A**		**B**	
	**Protonic cotton fibers**	**Deuterated cotton fibers**	**Protonic cotton fibers**	**Deuterated cotton fibers**
Sample no.	10	30	10	40
Classification value	0.5–1.5	1.51–2.5	0.5–1.5	1.5–2.5
Prediction value	1.04–1.29	1.35–2.49	1.46–1.70	1.64–2.09
Recognition no.	10	27	7	40
Recognition rate	100%	90%	70%	100%

**Table 5:** Classification result of partial least squares regression (PLS) models [cross-validation, A: high deuteration rate (20–60%) fibers and protonic fibers; B: high and low deuteration rate (1–60%) fibers and protonic fibers].

**Sample**	**A**		**B**	
	**Protonic cotton fibers**	**Deuterated cotton fibers**	**Protonic cotton fibers**	**Deuterated cotton fibers**
Sample no.	10	30	10	40
Classification value	0.5–1.5	1.51–2.5	0.5–1.5	1.51–2.5
Prediction value	1.00–1.21	1.49–2.29	1.11–1.48	1.49–2.25
Recognition no.	10	29	10	39
Recognition rate	100%	97%	100%	97.5%

As shown in Table 5 and Figure 5, the optimized PLS models effectively identified the deuterated fibers with both high and low deuteration rates. In the PLS models for high deuteration rate fibers, the recognition and rejection rates were 100 and 97%, respectively. In the PLS models for 1–60% deuteration rate fibers, the recognition and rejection rates were still as high as 100 and 97.5%, respectively. The predicted values of the samples in both models were closely gathered near the specified values as can be seen from Figure 5. These results indicate that the PLS models could achieve fast and accurate identification of the deuterated fibers with wide deuteration rates. The convenient NIR identification supports the potential application of the deuterated fibers as anticounterfeiting in this specialty study.

## Conclusion

Cotton fibers with a variety of deuteration levels were generated through a chemical hydrogen–deuterium exchange treatment. The deuterated fibers were characterized by FTIR, braking tenacity test, enzymatic hydrolysis, and bacterial culture test. The results showed that the breaking tenacity of chemically deuterated fibers was slightly lower than the controlled protonic fibers, which may be caused by the structural damage during the exchange treatment. The deuterated fibers were more stable to enzymatic hydrolysis degradation due to the KIE. Also, the low-level deuterium released from the deuterated fibers stimulated the growth of *E.coli*. NIR with SMICA, PCR, or PLS modeling was investigated for fast identification of the deuterated fibers. The PLS model showed the best results, which accurately distinguished protonic fibers from deuterated fibers with a deuteration rate of 1–60%. The fast identification of the deuterated fiber will help broaden its potential application.

## Data Availability Statement

The raw data supporting the conclusions of this article will be made available by the authors, without undue reservation.

## Author Contributions

YS and WJ developed the the research hypothesis and the experiment design. YS, HB, YZ, and GH performed whole experiments. AR revised the English and discussion. The final manuscript is the end product of joint writing efforts of all authors.

## Funding

This study was supported by the National Natural Science Foundation of China (51903131), the Natural Science Foundation of Shandong Province (ZR2019QEM007 and ZR2020ME076), the Key Research and Development Program of Shandong Province (2020CXGC011101), the State Key Laboratory of Bio-Fibers and Eco-Textiles (Qingdao University) (ZKT16 and ZKT21), and the Special Foundation of Taishan Scholar Construction Program (ts20190932).

## Conflict of Interest

The authors declare that the research was conducted in the absence of any commercial or financial relationships that could be construed as a potential conflict of interest. The handling editor declared a past collaboration with the author AR.

## Publisher's Note

All claims expressed in this article are solely those of the authors and do not necessarily represent those of their affiliated organizations, or those of the publisher, the editors and the reviewers. Any product that may be evaluated in this article, or claim that may be made by its manufacturer, is not guaranteed or endorsed by the publisher.
